# Ileosigmoid Knotting: A Case Series

**DOI:** 10.7759/cureus.32003

**Published:** 2022-11-29

**Authors:** Timon M Sseruwagi, Catherine Lewis

**Affiliations:** 1 General Surgery, Kampala International University, Ishaka, UGA; 2 General Surgery, East Tennessee State University, Johnson City, USA; 3 General Surgery, St. Joseph’s Hospital Kitovu, Masaka, UGA

**Keywords:** gangrene, volvulus, bowel ischemia, intestinal obstruction, ileosigmoid knotting

## Abstract

Ileosigmoid knotting is a rare case of intestinal obstruction that later leads to bowel necrosis. This is more common in males than females and seen more in areas with increased rates of sigmoid volvulus. The main clinical presentations are abdominal distention, abdominal pain and tenderness, vomiting, and obstipation. Definitive diagnosis is challenging due to its rarity and overlapping symptoms with other more common abdominal conditions. Delay in diagnosis and management can lead to peritonitis, necrosis of the bowel, sepsis, and eventually septic shock. Surgeons should consider ileosigmoid knotting in cases of acute abdomen, as it requires fast decision-making and intervention for a good prognosis. We present two cases of ileosigmoid knotting presenting with acute abdomen requiring emergent laparotomy with resection of necrotic bowel.

## Introduction

Ileosigmoid knotting is a rare surgical emergency in which the ileum makes a knot with the sigmoid colon, causing a closed-loop intestinal obstruction and bowel necrosis [1]. Ileosigmoid knotting was first reported by Parker in 1845, and the first patient in Africa was reported by Burkitt in 1952. Later, Shepherd named the condition "ileosigmoid knotting" in 1967. It has a male predominance, with a peak incidence during the third and fifth decades of life [2,3]. It is commonly encountered in areas with increased rates of sigmoid volvulus [4]. Misdiagnosis is common due to its rarity, nonspecific radiographic findings, and similarities to sigmoid volvulus. The diagnosis is typically made intraoperatively [3-5]. The etiology is not well established. However, the most predominant etiological factors are a long sigmoid colon with a narrow pedicle, a long small bowel mesentery with a freely mobile small bowel, and ingestion of a high-fiber, bulky diet with an empty small bowel [2,3]. Other contributing factors include late pregnancy, Meckel diverticulitis with thick bands, and ileocecal intussusception [3]. A quick decision is necessary to attain a good prognosis. We present two cases of ileosigmoid knotting requiring urgent exploratory laparotomy.

## Case presentation

Case one

**Figure 1:**
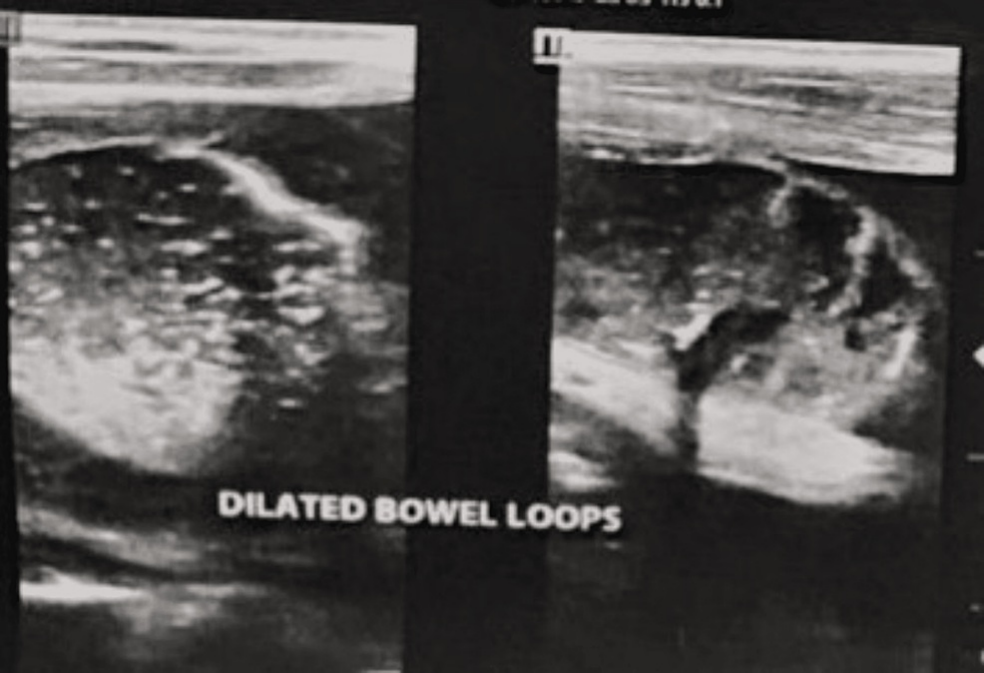
The patient's abdominal ultrasound demonstrated dilated loops of bowel with reduced peristalsis.

A 54-year-old male presented with a sudden onset of non-projectile, non-bilious vomiting and loss of appetite for two days, associated with abdominal distention. The review of other systems was unremarkable. On physical examination, he was sick-looking and afebrile. Vital signs were within normal limits. The patient was noted to have mild conjunctival pallor and skin tenting, but no pedal edema. The abdomen was mildly distended with tenderness in all four quadrants with rebound and guarding. Rectal examination revealed a swollen, non-tender, smooth, and rubbery prostate with no stool. Laboratory findings were significant for an elevated white blood cell count of 15.49 109/L. Sodium was decreased to 126 mmol/L (ref. 136-145 mmol/L) and chloride was 91 mmol/L (ref. 98-107 mmol/L). All other serum electrolytes were within normal limits. Abdominal ultrasound demonstrated dilated loops of bowel with reduced peristalsis (Figure 1).

**Figure 2:**
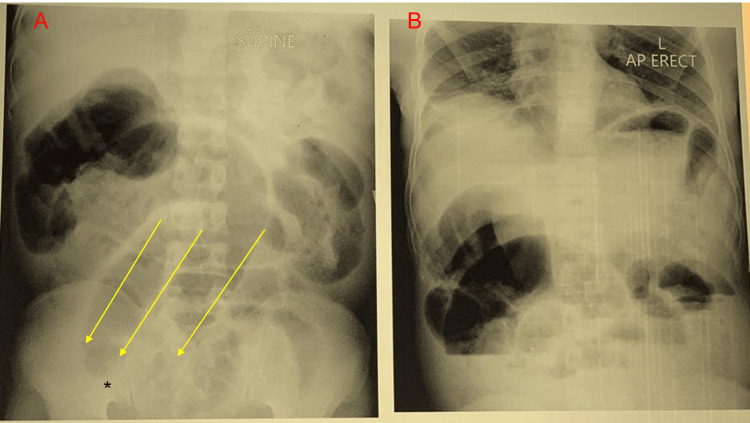
(A) Supine abdominal X-ray with the Frimann-Dahl sign (three linear shadows (yellow arrows)) converging to the site of obstruction (asterisk) and no rectal gas. (B) An erect abdominal X-ray demonstrated dilated loops of large bowel.

The abdominal x-ray demonstrated the Frimann-Dahl sign with the absence of rectal gas [6] (Figure 2).

**Figure 3:**
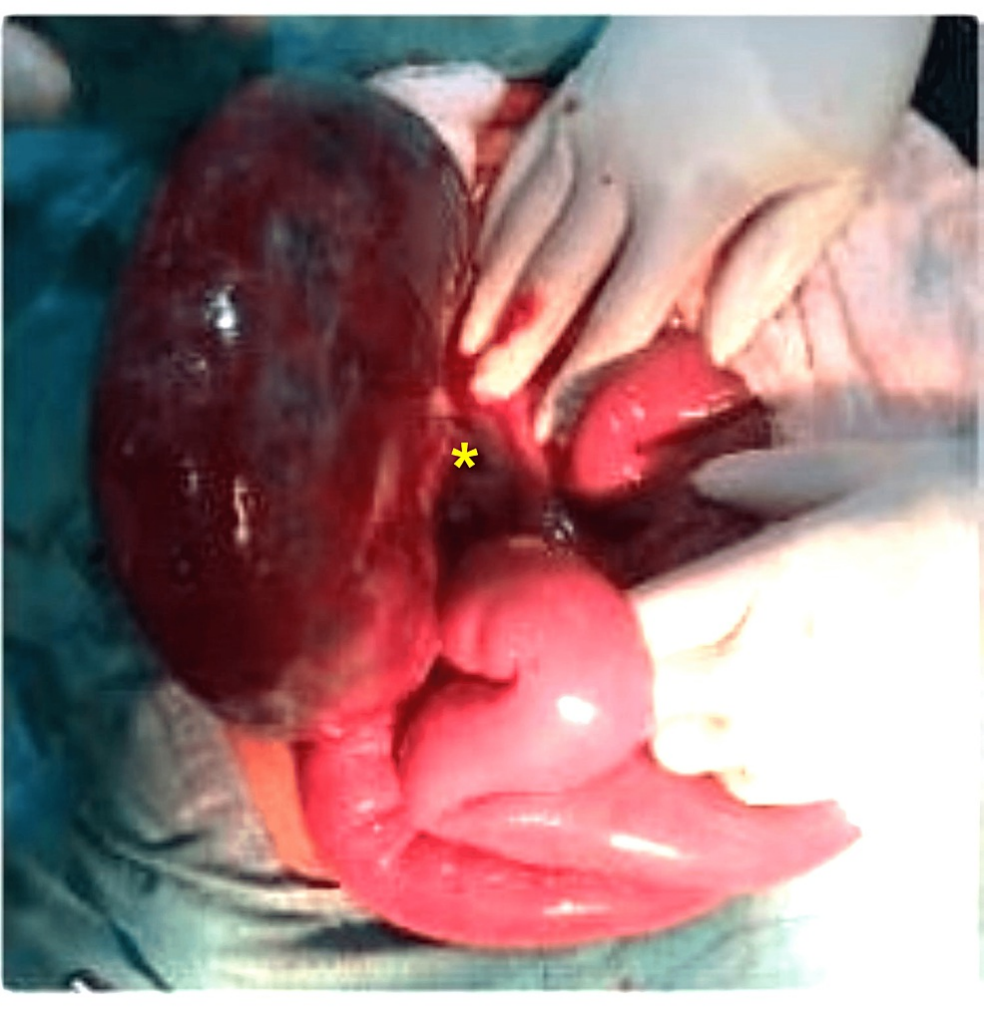
Intraoperative findings showed a dilated sigmoid colon with necrosis and knotting (indicated with the asterisk).

The patient was taken to the operating room emergently for an exploratory laparotomy. Upon exploration, the patient was noted to have knotting between the sigmoid colon and ileum. There was necrosis of the terminal ileum and the sigmoid colon (Figure 3).

The ileum was transected at the level of knotting. A sigmoidectomy was performed with a primary colorectal anastomosis. The necrotic small bowel was resected, and an ileostomy was placed. The patient tolerated the procedure without any immediate complications. The patient’s diet was advanced as tolerated. Postoperatively, the patient developed a superficial wound infection that was treated with intravenous antibiotics. The patient was discharged home on the 12th postoperative day. 

Case two

**Figure 4:**
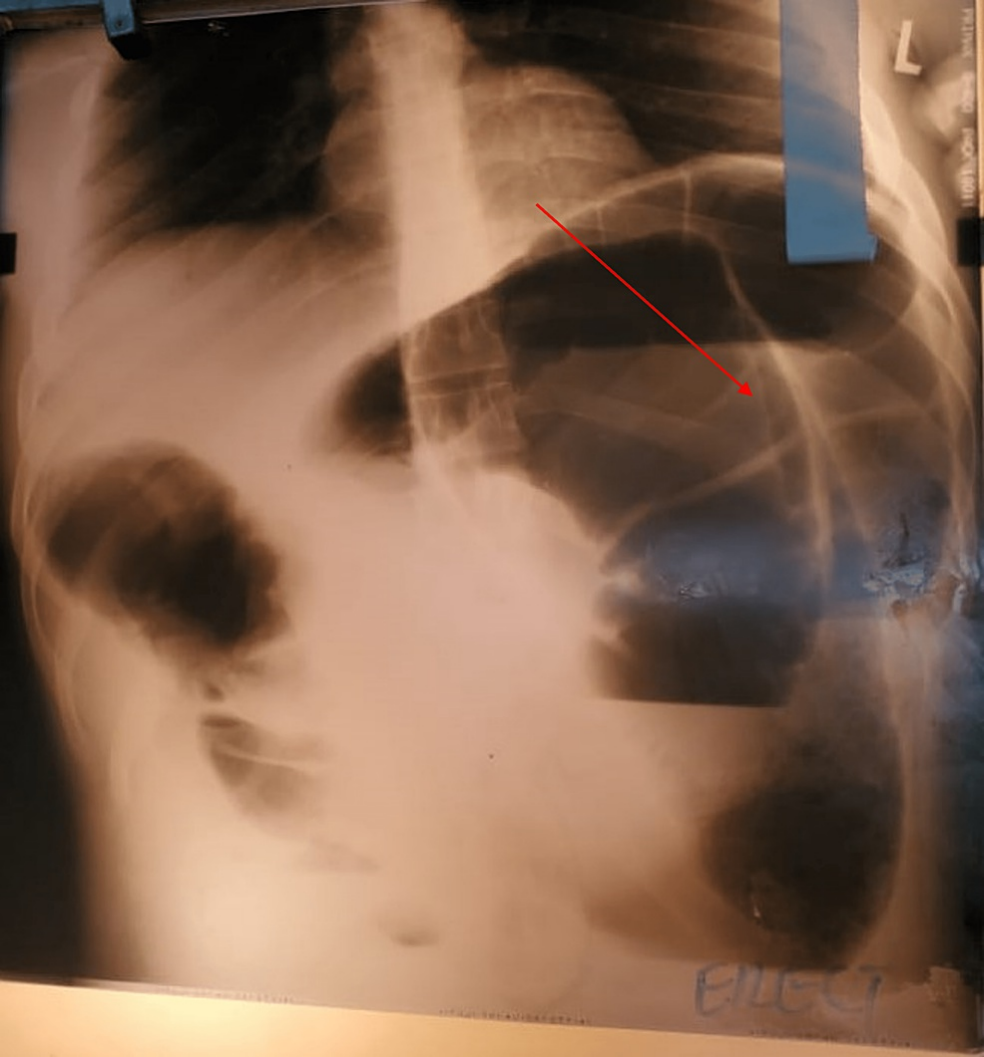
The patient's abdominal X-ray demonstrating sigmoid volvulus with a coffee-bean sign.

A 27-year-old male presented with a three-day history of colicky abdominal pain associated with bilious vomiting and abdominal distention. The last bowel movement was the night prior to the presentation. The abdominal examination noted hypertympanic bowel sounds, moderate distention, and generalized tenderness to palpation. There was associated rebound and guarding. The rectal examination did not demonstrate any masses, and the rectum was empty. The abdominal x-ray demonstrated findings consistent with volvulus (Figure 4).

**Figure 5:**
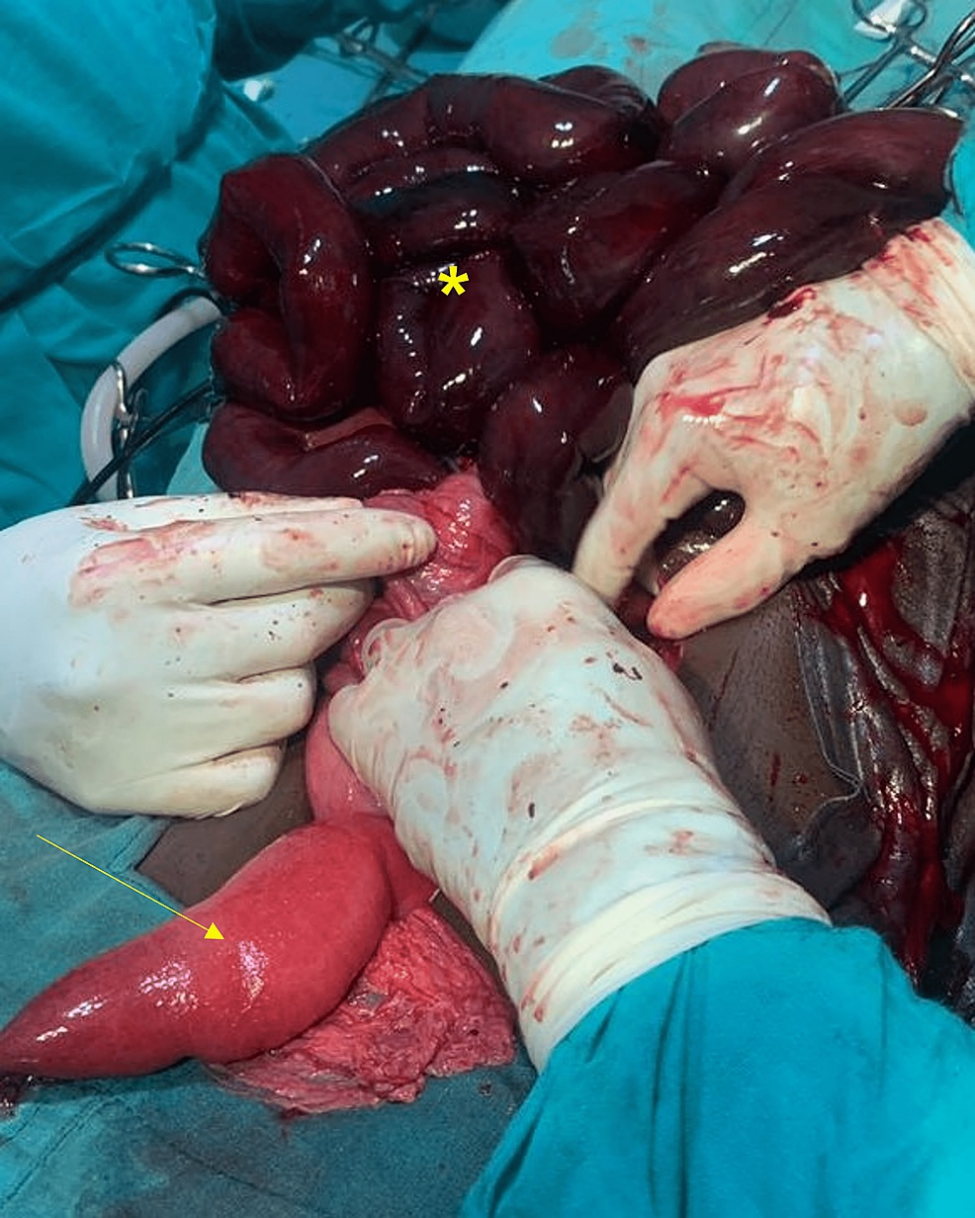
Intraoperative findings showed extensive necrosis of the ileum (asterisk) with a small area of normal jejunum (arrow).

Due to the examination and radiographic findings, the patient was taken emergently to the operating room for an exploratory laparotomy. On exploration, the patient was noted to have knotting of the sigmoid colon and ileum. There was extensive necrosis involving the sigmoid colon and ileum, extending to the distal jejunum (Figure 5).

The ileum was transected at the level of the knot at the base of the sigmoid colon. A sigmoidectomy was performed with a primary anastomosis between the descending colon and rectum. The necrotic small bowel was resected, and the distal jejunum was brought out as an ostomy. The patient tolerated the procedure well. The patient was started on oral feeds on postoperative day three. The patient was placed on a complex carbohydrate, high fat, and high protein diet consisting of multiple small meals per day. Postoperatively, the patient continued to have a decreased appetite and was unable to maintain an appropriate nutritional status. Due to financial constraints, the patient was not able to afford parenteral nutrition. The patient opted to be discharged to go to a regional referral center approximately three weeks after surgery. After discharge, the patient went home instead and died one week later.

## Discussion

Ileosigmoid knotting, also known as "double volvulus," is characterized by the formation of a knot (the ileum forms a knot with the sigmoid colon). It is less commonly encountered among white populations but more commonly seen in African, Asian, and Middle Eastern countries. Common causes include a long sigmoid with a narrow pedicle, a long small bowel mesentery with a freely mobile small bowel, and consumption of a bulky diet in the presence of an empty small bowel. Meckel’s diverticulum, internal herniation, malrotations, and postoperative adhesions are other rare predisposing factors [3,5]. It is categorized into four types: Type I - IV. In type I, the most common subtype, the ileum (the active component) makes a knot around the passive sigmoid colon. Type II is the opposite of type I, in which the sigmoid colon (the active component) makes a knot around the ileum. In Type III, the ileocecal segment makes a knot with the sigmoid colon. Type IV is undetermined, and it is impossible to determine what causes a knot with the other [1, 4]. A new classification system was described by Atamanalp et al. in 2009 based on preoperative and operative criteria that also correlate with mortality. Class-one patients are those with no risk factors. Class-two patients are those with no shock or bowel gangrene but who have the predisposing risk factors mentioned above. Class-three patients present with shock. Class-four patients have gangrene of the ileum or sigmoid colon. Class-five patients are those with both shock and ileum or sigmoid colon gangrene; those with both ileum and sigmoid colon gangrene are class 6 [3,4,7]. Both of our cases were Type I, in which the ileum was the active component. Both patients were also class-six, with necrosis of both the ileum and sigmoid colon.

The diagnosis of ileosigmoid knotting can be difficult preoperatively as there are no specific blood tests or radiographic imaging for the diagnosis. A computed tomography (CT) scan is the most preferred imaging modality. CT scan typically demonstrates a whirlwind sign with a median deviation of the cecum, the convergence of the left colon, and mesenteric vessels toward the whirlwind, with signs of ischemia [4,8]. Due to the economic status of our country, Uganda, X-rays and ultrasounds are more commonly used because CT scanning isn’t easily accessible and is relatively expensive. An abdominal X-ray usually shows a dilated sigmoid colon on the right side of the abdomen and multiple small intestinal air-fluid levels on the left side [9]. X-ray findings may also be similar to sigmoid volvulus as seen in our patients, in whom there is a large, dilated loop of bowel with a classic coffee-bean appearance (Figure 4) or the presence of the Frimann-Dahl sign (Figure 2) [6]. Barium or water-soluble contrast enemas will demonstrate an obstructive lumen. However, enemas are only indicated in patients without evidence of peritonitis, gangrene, or perforation [4]. Unlike sigmoid volvulus, endoscopic reduction is contraindicated, as ileosigmoid knotting is a surgical emergency, and attempted endoscopic reduction may cause injury or perforation [1-3,10]. Management should begin as soon as the diagnosis is suspected. In a few situations, the condition can resolve itself. Initial management includes fluid resuscitation, pre-operative antibiotics, and nasogastric decompression. Emergency laparotomy is necessary for an effective prognosis. There is no clear decision about the surgical procedure for ileosigmoid knotting [3,4]. Some authors advocate for untwisting the knot after enterotomy if both ileal and sigmoid bowel loops are viable [11]. Untwisting the knot can be difficult and risks the release of toxins and perforation. In cases of intestinal necrosis, it is advised to resect both the ileum and colon en bloc. A primary colorectal anastomosis and/or primary enteroenterostomy are feasible if both local and general conditions allow. Otherwise, an ileostomy or colostomy is required [4,10,11]. In each of our cases, untwisting of the knot was not possible, and a primary colorectal anastomosis with a small bowel ostomy was performed.

## Conclusions

Ileosigmoid knotting is a rare condition with a high mortality rate if not managed early. Timely intervention is required to avoid the development of peritonitis and gangrene. A high index of suspicion is necessary for patients who present with an acute abdomen, suspicious radiographic findings, and concerns for bowel ischemia and gangrene.
